# Implementation of Preoperative Very Low‐Calorie Diets in Preparation for Metabolic and Bariatric Surgery

**DOI:** 10.1155/jnme/7371866

**Published:** 2026-04-25

**Authors:** Tala Abedalqader, Annabella R. Strathman, Alberto Migliorini, Leonardo Garcia Cerecedo, Nour El Ghazal, Wah Yang, Adrian Dan, Manpreet S. Mundi, Omar M. Ghanem

**Affiliations:** ^1^ Department of Surgery, Mayo Clinic, Rochester, Minnesota, USA, mayo.edu; ^2^ Mayo Clinic Alix School of Medicine, Mayo Clinic, Rochester, Minnesota, USA, mayo.edu; ^3^ Department of Metabolic and Bariatric Surgery, The First Affiliated Hospital of Jinan University, Guangzhou, China, jd120.com; ^4^ Department of Surgery, Summa Health System, Northeast Ohio Medical University, Akron, Ohio, USA, neomed.edu; ^5^ Division of Endocrinology, Diabetes, Metabolism, and Nutrition, Mayo Clinic, Rochester, Minnesota, USA, mayo.edu

## Abstract

Very low‐calorie diets (VLCDs) have been commonly implemented prior to metabolic and bariatric surgery (MBS) with the goal of shrinking left liver lobe size and reducing intra‐abdominal mesenteric fat. Through this manuscript, we aimed to review the available literature discussing the types of VLCDs, their mode of implementation, preoperative duration, and the intraoperative and postoperative measures. A total of 103 articles was used in the generation of this review, including 3402 MBS candidates across the studies. VLCD was shown to induce significant preoperative weight loss, reduction in body fat mass, glycemic improvement, and decrease in proinflammatory makers. Liver volume reduction of 5%–23% and abdominal wall circumference reduction of 2.0–8.5 cm were noted following short regimens of VLCD, leading to improvement in perceived intraoperative difficulty. Postoperatively, metabolic improvements and reduction in perioperative complication rates were observed in some studies. In conclusion, VLCD demonstrated potential preoperative, intraoperative, and postoperative improvements in patients undergoing MBS.

## 1. Introduction

Global estimates have seen a steady increase in the incidence and prevalence of obesity over the years [1]. This increase has also been accompanied by a growth in the number of anti‐obesity interventions, including pharmacological advances and surgical procedures [2, 3]. Metabolic and bariatric surgery (MBS) has been consistently proven to be a safe and highly effective treatment of obesity and its associated medical conditions, leading to reductions in overall healthcare costs, morbidity, and mortality [4–7]. Currently, the Roux‐en‐Y gastric bypass (RYGB) and the sleeve gastrectomy (SG) comprise the two most commonly performed MBSs both in the United States and worldwide [3].

Patients are carefully selected for bariatric surgery based on several criteria, including body mass index (BMI) (> 30 kg/m^2^), the presence of obesity‐related medical conditions, and patient motivation [8]. Preoperative optimization is recommended to improve postoperative surgical outcomes and mortality. Lifestyle changes, including diet and exercise, have been utilized in the preoperative period to induce weight loss [9–11]. While longer diet regimens prior to bariatric surgery have been discouraged due to the adverse potential of hindering patient motivation and delaying necessary, life‐saving bariatric surgery, shorter regimens have been adopted with great success [9].

Restrictive diets, such as low‐calorie diet (LCD, ≤ 1200 kcal/day) and very low‐calorie diets (VLCDs, ≤ 800 kcal/day), have been recommended for 2–4 weeks prior to bariatric surgery. The Enhanced Recovery After Surgery (ERAS) Society guidelines have advocated for the use of restrictive diets to induce significant preoperative weight loss, leading to favorable anatomical changes and reducing intraoperative complexity [11]. Additionally, LCDs and VLCDs have demonstrated significant metabolic enhancements, potentially leading to improved cardiovascular health and lower postoperative complications [9–11]. While they are widely adopted, the existing literature presents conflicting data on the effect of VLCDs on surgical outcomes. We aimed, through this narrative literature review, to provide a comprehensive review on the implementation of VLCD before MBS, with a focus on clinical outcomes.

## 2. Methods

A structured methodological approach was used to conduct this narrative review paper. An extensive literature review was performed by the authors of this manuscript in three phases: identifying relevant research questions, performing the literature review, and reporting the most relevant findings. A narrative review design was selected due to the heterogeneity of available studies with respect to VLCD regimens, intervention duration, surgical procedures, and reported outcomes. Tasks were coordinated by all the involved authors. Databases, including PubMed, Scopus, and ScienceDirect, were searched from September to December 2025, identifying relevant articles that address the use of VLCD in the perioperative bariatric surgery setting and its physiological or clinical outcomes from inception to December 2025 according to our keywords: “very low‐calorie diet,” “very low‐energy diets,” “metabolic and bariatric surgery,” “preoperative optimization,” “intraoperative outcomes,” and “postoperative bariatric outcomes.” Retrospective studies, prospective studies, meta‐analyses, and systematic reviews reporting outcomes in English were included. Abstracts, non‐English texts, and articles that did not include our relevant search words were excluded. Manuscripts were then screened, and the final selection of articles was determined through review and consensus among all authors, with expertise and guidance provided by the senior authors and corresponding author. Given the nature of this review, registration in PROSPERO, an international prospective register of systematic review protocols, was not pursued.

## 3. Results

**FIGURE 1:**
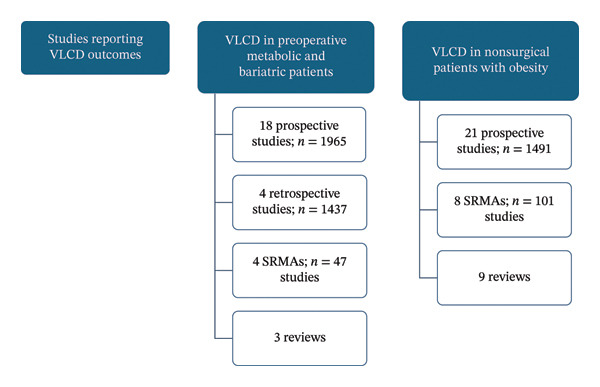
Types of studies used in the generation of the review. Abbreviations: VLCD: very low‐calorie diet, SRMAs: systematic review and meta‐analysis.

A total of 103 articles were used in the generation of this review; 67 studies, including prospective studies, retrospective studies, reviews, and meta‐analyses, reported outcomes of VLCD (Figure 1). Of those studies, 29 reported preoperative and/or postoperative outcomes of VLCD, encompassing a total of 3402 MBS candidates. Fifteen types of VLCD regimens were described across the studies.

### 3.1. Types, Indications, and Contraindications of VLCD

VLCDs, defined as nutritionally complete regimens providing ≤ 800 kcal/day, are used in the preoperative management of patients undergoing bariatric surgery. VLCDs are implemented in various formats, including mixed intake protocols (solid food, shakes, soups, and vegetables), liquid‐based protocols (e.g., soups, shakes, and yogurt), and ketogenic adaptations such as very low‐calorie ketogenic diets (VLCKDs), which restrict carbohydrates to ≤ 30 g/day and promote nutritional ketosis. Macronutrient compositions typically emphasize high protein (60–101 g/day), moderate to low fat (10–28 g/day), and controlled carbohydrate intake (50–111 g/day), with supplementation of essential micronutrients [12–18]. Although VLCDs are traditionally defined as ≤ 800 kcal/day, some structured programs slightly exceed this threshold and were classified as VLCDs in the original studies; thus, these terminologies were retained for consistency. Table 1 outlines the different types of VLCDs described in the literature.

**TABLE 1:** Types and contents of VLCD regimens.

Study, diet name	Calories/day	Macronutrient profile	Dietary components	Duration
González‐Pérezet al. [13], VLCD	800 kcal	P: 68 g F: 40 g C: 40 g	9.5 g fiber 478 mg sodium 1.7 mg potassium 838 mg phosphorus	6 weeks

Kumar et al. [12], VLCD	800 kcal	P: 60–80 g F: 10–15 g C: 50–70 g	Protein supplements Soups Vitamin and mineral supplements	4 weeks

Van Nieuwenhove et al. [16], Optifast	800 kcal	P: 70 g F: 15 g C: 100 g	5 shakes	2 weeks

Contreras et al. [19], Optifast	800 kcal	P: 72.8 g F: 8.3 g C: 93.6	30 g fiber 4 sachets Broth and non‐calorie beverages	3 weeks

Schwenger et al. [20], Optifast	900 kcal	P: 90 g F: 30 g C: 67.2 g	4 shakes Non‐calorie drinks Jello and stock < 10 cal/serving	3 weeks

Sivakumar et al. [21], Optifast	800 kcal	P: 80 g F: 18 g C: 72.8 g	4 shakes, bars, or soups Small bowl of low‐starch vegetables with olive oil	2 weeks

Lange et al. [14], BCM Diät	≤ 913 kcal	P: 75 g F: 28 g C: 80 g	1 Soup 2 Shakes 1 Bar	2 weeks

Lange et al. [14], Optifast	≤ 841 kcal	P: 58 g F: 19 g C: 111 g	1 Soup 4 Shakes	2 weeks

Schouten et al. [18], Prodimed	650 kcal	P: 101 g F: 16 g C: 12 g	Sachet of oat flakes Sachet of hot/cold drink Sachet of soup/omelet Sachets of dessert Prespecified vegetable quantities	10 days

Schouten et al. [18], standard VLCD	647–657 kcal	P: 81–86 F: 21–25 C: 20	200 g lean quark Prespecified vegetables Lean meat, fish, or eggs 1.5 L clear fluids Multivitamin tablets	10 days

Faria et al. [15], liquid VLCD	≤ 746 kcal	P: 78 g F: 20 g C: 67 g	Skim milk with whey powder Coconut water Soup Yogurt	2 weeks

Albanese et al. [17], VLCKD	≤ 700 kcal	P: 1.4 g/kg IBW F: 15–20 g C: ≤ 30 g	Protein shakes for breakfast and dinner Protein + 200 g vegetables for lunch Vitamin and mineral supplements	3 weeks

Erdem et al. [22], VLCKD‐SDM	10–12 kcal/kg/day	P: 16–18 g/portion F: 0.9–9.6 g/portion	Societa Dietetica Medica products containing 60–224 kcal/portion 0.1–12 g of fiber 2‐3 L of water Individualized nutritional supplements	2 weeks

Leonetti et al. [23], VLCKD	560–595 kcal	P: 72–80 g F: 23–24 g C: 15 g	8‐9 scoops of ketogenic powder Multivitamin, mineral, and omega 3 supplementation > 500 g of vegetables, up to 20 g of olive oil allowed	10 days

Leonetti et al. [23], VLCD	810 kcal	P: 80 g F: 30 g C: 55 g	Semi‐skimmed milk, low‐fat yogurt, or unsweetened orange juice 150–200 g lean meat or fish > 500 g of vegetables, 200 g of fruit 100 g of cheese 20 g of olive oil allowed	10 days

*Note:* Only diets used preoperatively in patients undergoing metabolic and bariatric surgery and with complete descriptions provided in the original studies are listed. C: carbohydrates, F: fats, and P: proteins.

Abbreviations: IBW, ideal body weight; SDM, Societa Dietetica Medica; VLCD, very low‐calorie diet; VLCKD, very low‐calorie ketogenic diet.

Indications for VLCD include obesity (BMI ≥ 30 kg/m^2^ or ≥ 35 kg/m^2^ with comorbidities) and the need for preoperative weight reduction. VLCD regimens are particularly utilized in patients with higher classes of obesity who are scheduled for bariatric surgery, with a recommended duration of 10 days to 6 weeks depending on the protocol and surgical timeline. This approach facilitates rapid weight loss within a short period, allowing for preoperative optimization and rehabilitation prior to MBS [19, 24, 25]. Contraindications include severe hepatic or renal dysfunction, coronary artery disease, heart failure, pregnancy, and lactation. Ketogenic variants of VLCD (VLCKD) are further contraindicated in patients with type 1 diabetes mellitus, arrhythmias, autoimmune diseases, galactosemia, patients using diuretics, and at extremes of age [24–26].

### 3.2. Physiological Effects of VLCD

The physiologic mechanisms and outcomes of VLCDs, including weight loss, changes in body composition, and metabolic changes, have been heavily studied. Many of the impacts are intrinsically linked to the weight loss seen among patients on VLCDs, although alternative proposed mechanisms exist in the literature.

#### 3.2.1. Weight Loss and Body Composition

The efficacy of VLCDs in inducing substantial short‐term weight loss is well established. The predominant mechanism is a marked caloric deficit leading to enhanced mobilization and oxidation of body fat stores [27, 28].

Across studies, VLCDs consistently yielded greater initial weight loss than other dietary strategies, depending on duration and caloric restriction intensity [29–33]. Greater caloric restriction correlates with more pronounced reductions in total body fat. VLCD leads to very rapid weight loss initially, followed by reduction at a rate of 1–4 kg per week [20, 34]. In a trial by Seimon et al., patients undergoing severe calorie restriction lost 3.1% of their body weight after 1 week compared to 1.6% in the moderate restriction group and 7.0% vs. 3.4% after 4 weeks, respectively, indicating a steeper initial decline that gradually plateaued over time [32]. Similar results were shown by Cicero et al.’s cohort, which had greater weight loss at 4 weeks compared to 8 and 12 weeks [35]. Studies examining VLCD use in MBS candidates have demonstrated weight loss of 3.6%–14.2% as early as 8 days after initiation, correlating to 4.5–9.1 kg and 1.6–4.9 kg/m^2^ reduction in weight and BMI, respectively [14, 16–22, 36–39]. Thus, these findings underscore the utility of VLCD as an effective intervention for rapid preoperative weight loss.

Beyond weight loss, VLCD has also been shown to result in significant changes in body composition, including reduction in fat mass of 1.6%–6.2% [14, 19, 21, 22, 37, 38] and decrease in weight circumference of 2.0–8.5 cm [15, 19, 23, 38, 40, 41]. Importantly, these changes have been observed as early as 1 week after VLCD and carry significant implications in preoperative patients relating to cardiometabolic risk improvements [42]. Despite these advantageous changes, concerns persist regarding loss of lean body mass during VLCDs [43–45]. The extent of lean‐mass reduction varies by age, sex, and baseline adiposity, with older adults, females, and individuals with lower baseline fat mass most affected [32, 44]. Resistance training and high‐protein supplementation can mitigate these effects [44, 46], and reductions in lean mass do not necessarily translate to diminished strength, particularly among women [32].

#### 3.2.2. Glycemic Control

VLCDs substantially improve glycemic control, particularly in individuals with type 2 diabetes mellitus (T2DM). The counterpoint study by Lim et al. was seminal in demonstrating reversal of T2DM through an 8‐week VLCD, showing significant reductions in fasting plasma glucose, insulin, and triglycerides, along with a 30% reduction in intrahepatic and intrapancreatic lipid content [47, 48]. A study by Saiyalm et al. showed that, in patients following a 12‐week VLCD regimen, significant glycemic improvements were apparent as early as 1 week following initiation. This was attributed to reduction in hepatic and pancreatic fat, leading to enhanced β‐cell function (BCF) and increased insulin sensitivity [49]. Subsequent trials confirmed these findings and extended them to patients with long‐standing T2DM, with 35.6% maintaining normoglycemia at 24 months [50, 51]. Factors such as disease duration and patient age were found to influence the likelihood of glycemic remission [48, 50, 52]. Systematic reviews have consistently shown significant reductions in hemoglobin A1c (HbA1c) and fasting glucose levels with VLCDs [48, 51, 53–55].

While the impacts on weight control are well‐established, the impact of VLCDs on BCF and insulin resistance (IR) is more variable. Some studies demonstrate improvements in both [47, 50, 55], whereas others show no significant effect [30, 50, 56, 57]. Anyiam et al. attributed these discrepancies to heterogeneous methodologies, differing VLCD durations, and small cohort sizes reported in the current literature [52].

In patients with T2DM, long‐term (> 8 weeks) VLCD has consistently demonstrated significant improvement in BCF across the literature, whereas short‐term regimens were more varied in their effectiveness [52]. IR, however, was found to have similar improvement regardless of T2DM status, with the most significant impact being on hepatic IR compared to whole‐body IR, which showed varied improvement [52, 58]. It has been suggested that the efficacy of VLCD to improve glycemic control is dependent on the patient’s capacity for beta cell recovery, which may explain these discrepancies [40, 48, 52, 55, 59]. Various mechanisms have been proposed to explain the impact of VLCDs on IR, including the improvement of oxidative stress [53]; however, little consensus exists in the literature.

#### 3.2.3. Inflammatory Markers

Reductions in proinflammatory cytokines such as interleukin‐2 (IL‐2), IL‐6, IL‐11, IL‐12, interferon gamma (IFN‐γ), tumor necrosis factor alpha (TNF‐α), and C‐reactive protein (CRP) following VLCD regimens have also been observed across multiple trials, with changes noted starting at 1 week and persisting for several weeks [19, 39, 53, 58, 60–62] up to 6 months [53, 62]. Meta‐analyses corroborate significant decreases in IL‐6 and CRP, with greater variability in TNF‐α response [63]. The decrease in proinflammatory markers was found to be greatest in patients who achieved > 5% total weight loss [63], as well as patients with altered baseline metabolic profiles [53]. Various mechanisms have been proposed to explain the improvement in inflammatory markers seen using VLCDs, including decreased oxidative stress [53, 62], improved IR [64], and decreased amount of adipose tissue in the body [53].

#### 3.2.4. Lipids

Improvements in lipid profiles have been observed as early as 1 week following VLCD initiation. Several studies have demonstrated significant reductions in total cholesterol, low‐density lipoprotein (LDL), high‐density lipoprotein (HDL), and triglyceride levels in patients with obesity after 1–8 weeks of VLCD [14, 35, 53, 54], whereas others observed no change [65, 66].

The National Lipid Association and other studies note that this variation may depend on the content of the VLCD, suggesting that LDL decreased more commonly following low‐saturated fat diets and even increased in diets consisting primarily of protein [67, 68]. However, other studies have shown no association between diet composition and LDL trends, suggesting that LDL response may rely on individual‐level differences between patients, such as APOE4 status [69, 70]. Notably, VLCDs may shift LDL particle size distribution toward larger, less atherogenic forms and reduce very low‐density lipoprotein (VLDL) size without changing total LDL particle number [53].

These findings suggest that short‐term VLCDs generally produce favorable or neutral lipid effects, supporting the metabolic safety of preoperative VLCDs while underscoring the potential value of individualized dietary composition and lipid monitoring in high‐risk patients.

### 3.3. Intraoperative Outcomes

Obesity introduces several challenges to any surgical intervention [71], which include but are not limited to anesthesiologic issues, where narrowing of the airways from the redundant tissue makes intubation more difficult [72]. The reduced functional residual capacity (FRC) and chest wall compliance also increase the risk of rapid desaturation after induction of anesthesia [73]. Additionally, access to the abdominal cavity and creation of an adequate surgical field may be hindered by the thickness of the abdominal wall [74]. During MBS, the enlarged liver parenchyma associated with obesity can obstruct the visual field potentially contributing to parenchymal injury when accessing the abdominal cavity, and thickened mesentery can make mobilization of the bowel loops more challenging [37]. In addition, higher fat content, increased BMIs, and larger waist circumferences increase the softness and fragility of those structures, causing higher rates of complications and conversions from minimally invasive surgery (MIS) to open approach [75].

The advent of laparoscopic or robotic minimally invasive approaches has been a turning point for the development of MBS in recent decades. In addition, anesthesiologic and clinical care measures, such as intraoperative positioning recommendations and enhanced recovery after bariatric surgery (ERABS), have been adopted to improve surgical outcomes. Among these ongoing improvements, preoperative diet management is a relevant factor, as it allows detection and correction of micronutrient deficiencies [71, 76] and has demonstrated a net reduction in complications [77, 78]. Owing to the operative challenges faced with increased adiposity, preoperative weight loss strategies have been implemented to minimize those complications. Studies have consistently demonstrated that significant preoperative weight loss leads to shorter and less challenging operations. For instance, Liu et al. reported lower intraoperative blood loss in patients with excess weight loss of > 1% [79]. In a retrospective study by Alami et al., patients who lost > 10% of their weight before RYGB had significantly reduced operative time (220.2 versus 257.5 min; *p* = 0.0084) when compared to patients losing less or no weight at all [80].

Given the reductions in weight and visceral fat demonstrated with VLCDs and the resultant anatomic variations, this diet regimen has been implemented in preoperative optimization programs before abdominal operations, including bariatric and nonbariatric procedures [16, 81–86]. One of the major changes seen with VLCD is reduction in liver size, with studies showing reductions of up to 5%–23% following this regimen visualized on various imaging modalities, most commonly computed tomography (CT) reported in systematic reviews, as well as ultrasound and magnetic resonance imaging (MRI) [19, 37, 82, 87, 88]. This is of particular relevance, as it improves visualization during the operation, potentially leading to lower complications and short operating times. Fris et al. reported that, in patients undergoing laparoscopic gastric banding, no large left lobe of the liver was encountered at surgery nor caused any problem in any patients with successful preoperative weight loss [37]. Acute caloric restriction with VLCD also results in significant visceral fat reduction, which has been inversely correlated with operative time [15].

Studies by MacCormick et al. and Chowdhury et al. described reduced liver volume, less technical difficulty, and lower blood loss in patients who followed VLCD before upper gastrointestinal and other abdominal surgeries [81, 84]. Similar results have also been demonstrated in bariatric procedures, but with little consensus in the literature. A randomized clinical trial of patients across five European countries compared the effect of VLCD versus no dietary restriction 2 weeks before MBS. Significant preoperative weight loss was achieved in the VLCD group when compared to controls (−4.9 kg vs. −0.4 kg, *p* < 0.001), and surgeons’ perceived difficulty of the operation was lower in the VLCD group. However, objective measures such as operative time, intraoperative blood loss, and complications were not significantly different [16]. Several studies have also reported no difference in operative duration with VLCD [83, 85]. In contrast, others have demonstrated opposing findings, with reduced operative times following VLCDs [15, 86]. While VLCD reliably reduces liver volume and may subjectively facilitate operative exposure, current randomized evidence does not demonstrate a corresponding reduction in objectively measured intraoperative or perioperative outcomes.

### 3.4. Postoperative Outcomes

While VLCD confers several preoperative and intraoperative benefits, the current literature shows conflicting data regarding its effect on weight loss and complications following bariatric surgery. Preoperative optimization of bariatric surgery is mainly aimed at weight reduction to reduce surgical complexity, and significant weight loss prior to MBS has been shown to be additive to postoperative weight loss [89, 90]. A Scandinavian Obesity Registry Study supported these results, demonstrating a correlation between preoperative weight loss and sustained weight reduction after RYGB; patients who lost 8.6% or more of their weight preoperatively achieved up to 15.2% higher weight loss postoperatively when compared to patients losing less weight [90]. While the correlation between preoperative VLCD‐induced and postoperative weight loss remains unclear, several studies have similarly reported greater immediate postoperative weight loss among patients who achieved substantial preoperative weight reduction of > 8%, suggesting that the magnitude of preoperative weight loss, rather than the type of diet itself, may drive this effect [24, 91, 92]. Conversely, other studies have found no significant difference in short‐term (< 1 year) [16, 18, 19, 36] or long‐term (up to 5 years) [83, 86] postoperative weight loss between patients who underwent a preoperative VLCD and those who followed a standard diet or no dietary intervention.

Although weight‐related outcomes are conflicting, VLCD has demonstrated metabolic improvements, potentially leading to better outcomes. Patients with obesity often present with metabolic abnormalities, including IR, hyperglycemia, and hypercholesterolemia, which subsequently lead to worse surgical outcomes [82]. Schwenger et al. conducted a prospective study in patients with metabolic dysfunction–associated steatotic liver disease (MASLD) undergoing MBS and demonstrated significant decrease in BMI (−2.25 kg/m^2^), insulin (−148.8 pmol/L), HbA1c (−0.26%), and cholesterol (−0.89 mmol/L) after the implementation of VLCD [20]. Similar results were also reported by several studies [82, 86, 93], with VLCD showing greater reductions in HbA1c (−0.24%) following RYGB up to 5 years postoperatively [86]. Interestingly, Lips et al. described improvements in glycemic indices after VLCD in patients with T2DM that were comparable with RYGB [93], and similar results were corroborated by Saiyalam et al. [49]. This highlights the potential utility of VLCD in the optimization of patients with high‐risk metabolic features before bariatric interventions.

Building on the metabolic improvements associated with VLCD, Nieuwerhove et al. reported a reduction in 30‐day postoperative complications (5.8% in VLCD vs. 13.2% in control group), which they attributed to presumed improvements in glycemic control, although these parameters were not directly assessed [16]. Despite such observations, evidence regarding the effect of preoperative VLCD on postoperative complications remains limited and inconsistent. A systematic review by Simancas‐Racines et al. reported a significant reduction in perioperative complications (odds ratio of 0.59, *p* = 0.03) and slightly shorter hospital stay (mean difference of −0.17 days, *p* = 0.0001) with VLCD compared to LCD [85]. Albanese et al. further compared a standard VLCD with its ketogenic variant (VLCKD), noting improved hemoglobin levels (13.1 vs. 12.7 mg/dL), drainage output (141.2 vs. 190.7 mL), and requirement for length of stay beyond 3 days (2.8% vs. 10.4%) following VLCKD, suggesting that even within VLCD protocols, nutritional composition may influence immediate postoperative outcomes [17]. These findings, however, were limited by the small sample of patients included, and further prospective studies with larger sample sizes are needed to confirm these results.

In contrast, the majority of studies have found no significant difference in long‐term postoperative outcomes between VLCD and non‐VLCD groups [18, 19, 26, 83, 87, 88, 94]. In fact, Chakravartty et al. demonstrated significantly lower levels of mature collagen type I in skin biopsies of patients following VLCD regimens compared to standard diet, suggesting a potentially adverse effect of the intervention. Given that collagen type I is central to wound tensile strength, this raises theoretical concerns regarding wound and anastomotic healing. However, since clinical outcomes, such as wound dehiscence, anastomotic leak, and hernia occurrence, were not assessed, its clinical relevance remains uncertain [87]. Only one study reported mortality rate [16], which was limited to 30 days after surgery and was not different between VLCD and non‐VLCD cohorts.

### 3.5. Challenges and Adverse Effects of VLCDs

Despite the benefits demonstrated with perioperative and short‐term use, long‐term adherence and weight loss have been challenging with rates of adherence to VLCD ranging from 61.8% to 93% [36, 82]. The variability of adherence has been attributed to differences in definitions and measurement methods among studies [36]. Additionally, a number of patient‐related factors may influence adherence, including potential adverse effects, tolerability of diet regimen, appetite changes, and increased hunger. VLCD can be associated with significant side effects ranging from transient neurological symptoms, such as headache or dizziness, to gastrointestinal disturbances including nausea, vomiting, constipation, loose stools, or gallstones, and electrolyte imbalances [24, 25, 81]. Frequent monitoring, shorter regimen durations, and inclusion of solid food may increase compliance with VLCD through prompt recognition and management of adverse effects and the mitigation of hunger sensation [36].

Although rodent studies note that caloric restriction can promote the development of beige fat within white adipose tissue (WAT) depots, human data do not support a similar effect [94, 95]. Barquissau et al. noted that in 289 individuals with obesity, 8‐week VLCD followed by 6‐month weight maintenance phase resulted in overall decreased browning of subcutaneous abdominal WAT although individual results were variable [96]. The discrepancy between animal and human responses may be due to differences in adipose tissue biology and the regulation of thermogenesis.

Long‐term use of VLCD also leads to a reduction in resting metabolic rate, total energy expenditure, and diet‐induced thermogenesis that exceeds what would be expected from loss of body mass alone. This phenomenon, known as adaptive thermogenesis, can amount to an extra 5%–15% decrease in resting metabolic rate compared to predictive value based on body composition [97] and may be related to degree of caloric restriction. Adaptive thermogenesis may partly be explained by the reduction in leptin and insulin leading to downregulation of substrate cycling in the skeletal muscle as well as indirect suppression of sympathetic‐thyroid axis [98]. In addition to metabolic rate, VLCDs are associated with a decrease in diet‐induced thermogenesis, which is typically ∼10%‐15% of total energy expenditure [99], further contributing to reduction in total energy expenditure. In a 20‐day VLCD study in ten young and nine older men, a decrease of 13% and 23.6%, respectively, was observed in the thermic effect of food over 4 h after a meal accounting for 25% of total daily energy needs was consumed [100]. These changes in energy expenditure can make continued weight loss harder, leading to weight plateau and even weight regain despite ongoing caloric restriction.

In addition to changes in energy expenditure, VLCDs can trigger a shift in appetite regulating hormones that collectively increase hunger and diminish satiety. One of the most pronounced changes is a sharp decrease in circulating leptin which is typically proportional to the loss of fat mass [99, 101]. The decline in leptin removes an important signal of energy sufficiency, thereby reducing central satiety tone and increasing appetite [99, 101, 102]. Similarly, gut‐derived anorexigenic hormones including peptide YY (PYY), glucagon‐like peptide‐1 (GLP‐1), and cholecystokinin (CCK) decrease in response to caloric restriction, further promoting hunger. Simultaneously, ghrelin, which is a gastric hormone that stimulates hunger, rises in response to caloric reduction. Studies have noted that these changes can persist beyond the active caloric restriction period, further creating a hormonal milieu favoring weight regain [101]. While adaptive thermogenesis is a well‐described response to caloric restriction, its effects are likely minimal over the short preoperative VLCD window of 2–6 weeks. However, understanding the physiological response to longer VLCD durations provides critical context that discourages prolonged use or delaying MBS to extend VLCD duration in pursuit of potentially greater benefits.

Careful preoperative assessment, counseling, and monitoring of patients prior to implementation of VLCDs are necessary to ensure appropriate adherence and prevention or management of side effects. In patients with T2DM, adjustment or cessation of antidiabetic medications, especially insulin, should be done shortly after the start of VLCD. Similar changes should be made in patients on antihypertensive medications. Patients who remain on medication regimens must be educated about the risk of hypoglycemia or hypotension, and routine monitoring of blood sugar levels and blood pressure should be advised [11, 49]. Additionally, it is important to focus on weight history and previous weight loss attempts, as Swager et al. noted that patients who cycled repetitious VLCD attempts without appropriate monitoring were more likely to experience complications and suboptimal weight loss [103].

## 4. Conclusion

The medical literature reports conflicting data on the utility and effectiveness of short‐term use of VLCDs prior to bariatric procedures. These inconsistencies may reflect heterogeneity in study design, patient populations, and perioperative management protocols, as well as the varying definitions of complications and duration of follow‐up across studies. VLCDs confer significant benefits in reducing liver volume and visceral fat, thus leading to better intraoperative visualization and lower surgical complexity. While preoperative VLCD regimens have shown no consistent effect on weight loss following MBS, their use has demonstrated substantial metabolic improvements, particularly in insulin sensitivity, yielding improved pre‐ and postoperative glycemic control and potentially leading to less complications. Their utilization is further supported to optimize individuals with obesity and metabolic disorders, including T2DM and hepatic steatosis. That said, patients should be assessed carefully before implementation of VLCD, and caution should be taken in patients presenting with hepatic, renal, and cardiovascular impairments. Additionally, careful monitoring by a dietitian throughout the regimen should be performed to effectively recognize and manage adverse effects and nutritional status before bariatric procedures. Although there seems to be a trend to more favorable outcomes with VLCD implementation as part of the preoperative MBS regimen, larger, prospective studies are needed to accurately elucidate their effects on objective intraoperative and specific long‐term postoperative outcomes.

## Funding

This research did not receive any specific grant from funding agencies in the public, commercial, or not‐for‐profit sectors.

## Conflicts of Interest

Adrian Dan is a proctor for Intuitive Surgical; Manpreet S. Mundi has received research grants to his respective institution from Nestle and NorthSea and serves on the advisory board for Baxter, Fresenius Kabi, Nutrishare, and Otsuka; Omar M. Ghanem is a speaker for Olympus and consultant for Intuitive and Medtronic. The aforementioned COIs did not impact the results or conclusion of this manuscript but have been disclosed due to potential relevance to clinical nutrition and metabolic and bariatric surgery. All remaining authors declare no conflicts of interest.

## Data Availability

No new data were created in this study.
